# Evaluation of the safety and efficacy of surgery for radio-recurrent prostate cancer: a systematic review and meta-analysis

**DOI:** 10.3389/fonc.2025.1674005

**Published:** 2026-01-16

**Authors:** Shengyu Zhu, Jianjiang Liu, Wei Zhong, Bin Shen, Huali Xu, Jiajing Ni

**Affiliations:** 1 Department of Radiotherapy, Shaoxing Second Hospital, Shaoxing, Zhejiang, China; 2 Department of Urology, Shaoxing People's Hospital, Shaoxing, Zhejiang, China; 3 School of Medicine, ShaoXing University, Shaoxing, Zhejiang, China; 4 Department of Cardiac Surgery, Shaoxing People’s Hospital, Shaoxing, Zhejiang, China; 5 Department of Urology, Shaoxing People’s Hospital, Shaoxing, Zhejiang, China

**Keywords:** prostate cancer, prostatectomy, radio-recurrent, recurrence, salvage, surgery

## Abstract

**Background:**

Surgery plays a critical role in managing radio-recurrent prostate cancer (PCa). This study aims to comprehensively review its effectiveness and associated severe complications.

**Methods:**

A thorough review of PubMed and EMBASE databases up to July 2024 was conducted, focusing on recurrence-free survival (RFS) with salvage surgery across various subgroups. Severe complications were also assessed using the Clavien-Dindo Scale (CDS). Survival curves were reconstructed using WebPlotDigitizer and a newly developed shiny application.

**Results:**

Forty-four studies were included, with 17 papers (2056 patients) contributing to survival curve reconstruction. Among 1654 patients treated with salvage surgery after eliminating duplicate cases, the median RFS was 63.9 months, with 2-, 3-, and 5-year rates of 65.6%, 59.3%, and 51.2%, respectively. Factors associated with better RFS included robot-assisted surgery [hazard ratio (HR):1.49, p < 0.001], lower rates of seminal vesicle invasion (SVI) (HR: 0.75, p = 0.006) and lymph node involvement (LNI) (HR: 0.74, p = 0.006), higher proportion of adjuvant androgen deprivation therapy (ADT) (HR: 2.96, p < 0.001), and higher values of pathological Gleason scores (GS) (≤7/≥8) (HR:1.30, p < 0.001). Severe complications (grade ≥ IIIa) occurred in 404 out of 2537 patients (15.9%, 95% CI: 14.5 to 17.4).

**Conclusions:**

This study comprehensively assesses complications and conducts a pooled analysis of RFS for salvage surgery in radio-recurrent PCa. Robot-assisted surgery, lower rates of SVI and LNI, adjuvant ADT, and higher proportions of pathological GS ≤7 appear promising as prognostic factors for RFS. However, confirming these findings will necessitate randomized controlled trials due to low levels of evidence and study heterogeneity.

## Introduction

Prostate cancer (PCa) is the most common non-cutaneous solid organ malignancy in the United States and ranks as the second leading cause of cancer-related mortality (1). Approximately one-third of localized prostate cancer patients undergo radiation therapy with curative intent (2). However, up to two-thirds of men experience biochemical recurrence (BCR) within 10 years following initial radiation therapy (3, 4). BCR was defined as a rise in their prostate-specific antigen (PSA) level of 2 ng/mL above the postradiotherapy nadir. Therefore, proper management of patients experiencing post-radiation recurrence is critical for radiation oncologists and urologic surgeons.

Despite 50% of recurrences being localized to the prostate, many patients undergo androgen deprivation therapy (ADT), thereby forfeiting the opportunity for cure. Recent guideline updates recommend observing these patients or enrolling them in clinical trials due to the nuanced and multidisciplinary approach necessary for their management (5). Patients confirmed with localized recurrence may consider salvage radiation therapy, ablative therapies, or radical prostatectomy (6). Despite the absence of high-level evidence and direct comparative studies versus ADT or repeat external beam radiation therapy (EBRT), re-irradiation — especially in the form of stereotactic body radiation therapy (SBRT) — has become a safe and well-tolerated treatment option for locally recurrent prostate cancer following primary radiotherapy, with a low incidence of severe toxicity (7).

In recent years, there has been renewed interest in salvage radical prostatectomy (sRP), with contemporary series demonstrating lower incidence rates compared to historical data, including robotic surgery (8). Considering the increasing use of non-surgical primary treatments and local therapies in prostate cancer management (9), the demand for curative salvage surgeries such as salvage robot-assisted radical prostatectomy (sRARP) may escalate. However, meta-analyses specifically addressing salvage surgical treatments for radiation-recurrent prostate cancer are rare, and comprehensive data on recurrence-free survival (RFS) and rates of severe complications remain insufficient.

In our recent meta-analysis (10), we analyzed RFS outcomes and toxicity profiles in patients with radio-recurrent PCa treated with salvage high-dose-rate brachytherapy (HDR-BT). This analysis revealed significant subgroup findings and summarized the incidence of adverse events (AEs). However, to date, there is a lack of comparable meta-analyses focusing on salvage surgery. Hence, the goal of this systematic review and meta-analysis is to assess the efficacy and complications associated with surgical interventions for radio-recurrent PCa.

## Materials and methods

### Research design

The evaluation protocol was prospectively registered in the International Prospective Register of Systematic Reviews (PROSPERO) and is publicly accessible under registration number CRD42024561208.

### Data source and searches

We conducted a meticulous and systematic literature search across two reputable electronic databases, Embase and PubMed, spanning articles from their inception to July 23, 2024. Full-text articles were independently screened for eligibility by two investigators. The search strategy utilized relevant terms: (radiation therapy OR radiotherapy OR radio-resistant OR brachytherapies OR radioresistant OR radio-recurrent OR brachytherapy OR radiorecurrent) AND (prostate OR prostatic) AND (Salvage OR Recurrence OR local failure OR recurrent OR resistant OR radiation failure OR relapse OR recrudescence OR biochemical failure) AND (prostatectomy OR salvage surgery OR prostatectomies OR robot) (see **Supplementary Table S1**). Additionally, reference lists of eligible studies underwent manual scrutiny for potential additional inclusions.

### Study selection and eligibility criteria

#### Inclusion criteria

1. Patients diagnosed with radio-recurrent prostate cancer.
2. Availability of quantitative data on either RFS or severe complications treated with surgery, with RFS curves demonstrating rates extending beyond two years.

#### Exclusion criteria

1. Clearly duplicated publications.
2. Articles lacking full-text availability.
3. Publications not written in English.
4. Studies where the Clavien-Dindo Scale (CDS) was not utilized to assess severe complications.

#### Inclusion criteria for RFS curve reconstruction

1. Meeting the aforementioned inclusion and exclusion criteria.
2. Presence of risk tables in the RFS curves.

#### Exclusion criteria for RFS curve reconstruction in subgroup analysis

1. Duplicate data.

Survival curve reconstruction was independently conducted by two investigators, with any discrepancies resolved through consensus.

### Data extraction

Two investigators independently employed a standardized data extraction form to collect data, resolving discrepancies through discussion. Patient characteristics data encompassed two main aspects: 1) Primary disease and treatment features; 2) Details of disease and treatment during the peri-salvage surgery period. Additionally, we extracted raw data coordinates and numbers at risk from original papers to reconstruct individual patient data (IPD) for RFS analysis. To mitigate the impact of duplicate reports, we will meticulously filter redundant data by considering the institutions of enrollment and enrollment periods, ensuring the accuracy of RFS and severe complications data.

### Data synthesis and analysis

The primary aim of this study is to evaluate RFS among radio-recurrent PCa patients treated with salvage surgery across diverse subgroups, with a secondary objective of assessing severe complications. Various definitions of RFS are observed in the literature, with the Phoenix criteria being the predominant standard. For this investigation, biochemical recurrence-free survival, failure-free survival, disease-free survival, and progression-free survival are considered synonymous with RFS. Complications will be evaluated using the CDS, where Grade ≥ 3a denotes severe events (11).

Survival data will be reconstructed by capturing screenshots of necessary RFS curves and risk tables from each publication. Raw data coordinates will subsequently be extracted using the semi-automated tool WebPlotDigitizer. IPD will be reconstructed using a novel application developed by Liu et al. (12), followed by the generation of survival curves using R (version 4.0.3). Incidence rates of complications will be summarized along with 95% confidence intervals (CIs). These rates and CIs will be calculated using a random effects model with logit transformation, as described by Nyaga et al. (13), and analyzed using STATA 14.0. Statistical significance will be determined using a two-sided test at a significance level (α) of 0.05, with results considered significant if they meet this criterion.

## Results

### Study selection and patient characteristics

Following removal of duplicate records, a total of 11,253 entries were retrieved from two databases. Initial screening of titles and abstracts, and subsequent exclusion of records not meeting inclusion criteria, resulted in retaining a final set of 133 records (see **Supplementary Figure S1**). A comprehensive review of full texts led to the inclusion of 44 studies (14–57). Curve data reading software was employed to extract 2-year or 5-year RFS rates from 30 papers (14–43) (see **Supplementary Table S2**). RFS curves were reconstructed for 17 studies (14, 18–21, 23–25, 29, 31–33, 36, 38, 40, 44, 45), with evidence of substantial duplication observed in 3 studies (32, 33, 45) (see **Supplementary Table S3**). Following exclusion of seven nearly completely duplicated studies (see **Supplementary Table S4**), a summary of severe complications from 22 studies (14–16, 18–22, 28, 45–57) was provided, noting partial duplication in 5 studies (28, 54–57).

**Table 1** presents patient characteristics related to primary disease and treatment from 17 studies contributing to the reconstruction of RFS curves. Publication dates ranged from 1998 to 2023, encompassing four prospective and the remaining retrospective studies. Patient enrollment spanned from 1963 to 2021, primarily from institutions in the United States, Australia, and Europe. Study sizes varied, with enrolled patients ranging from 25 to 414. Median pre-treatment PSA levels ranged from 5.4 to 14.5 ng/ml. Pre-treatment Gleason scores (GS) were outlined across studies, though data were often incomplete. The majority of patients received definitive treatment primarily via EBRT, supplemented by other modalities such as brachytherapy, and proton beam therapy.

**Table 1:** Primary disease and treatment characteristics.

First author	Year	Design	Inclusion time	Institutions	Patients (n)	Initial PSA (ng/mL, range)	Initial GS (%)		Primary treatment
							≤7	≥8	
Lama DJ (13)	2023	P	2004-2021	Prospective database (approval #00149)	78	NR	NR	NR	BT (40%)/EBRT (38%)/Proton beam therapy (10%)/cryotherapy (6%)/BT+EBRT (4%)
Ribeiro L (17)	2021	R	2007-2019	Guy’s Hospital (London, UK), Institut Mutualiste Montsouris (Paris, France), Imperial College Healthcare Trust (London, UK) and The Peter MacCallum Cancer Centre (Melbourne, Australia)	90	14.5 (2.7-78.3)	88	12	EBRT (62%)/High dose BT (4%)/Low dose BT (30%)/Cyberknife(1%)/EBRT+BT (2%)
Rajwa P (44)	2021	R	2007-2015	Medical University of Vienna/King Fahad Specialist Hospital/Jordan University Hospital/University Medical Center Hamburg-Eppendorf/Jikei University School of Medicine	214	NR	NR	NR	EBRT (78%)/BT (18%)/EBRT+BT (3.7%)
Nathan A (18)	2021	R	2012.1-2020.3	University College London Hospitals NHS Foundation Trust	49	NR	NR	NR	RT (71.4%)/BT (20.4%)
Martinez PF (open) (19)	2021	R	2004.8-2019.3	Hospital Italiano de Buenos Aires, Argentina	50	8.1 (6.1-10.8)	94	6	BT (14.5%)/3D EBRT (77.6%)/IMRT (7.9%)
Martinez PF (robot) (19)	2021	R	2004.8-2019.3	Hospital Italiano de Buenos Aires, Argentina	26	8 (7.2-11)	84.6	15.4
Marra G (20)	2021	R	2000-2016	18 tertiary referral centers in United States, Australia and Europe	414	NR	NR	NR	EBRT (64.5%)/BT (25.7%)
Quhal F (43)	2020	R	2007-2015	Medical University of Vienna/King Fahad Specialist Hospital/Jordan University Hospital/University Medical Center Hamburg-Eppendorf/Jikei University School of Medicine	214	NR	87.08	12.92	BT/EBRT/EBRT+BT/EBRT+intensity modulated RT/EBRT+three-dimensional conformal RT
Onol FF (22)	2020	R	2008-2018	Advent Health Global Robotics Institute	94	NR	NR	NR	EBRT (41.5%)/intensity modulated radiation (16%), proton beam radiation (3.2%)/BT (24.5%)/EBRT+BT (14.9)
Devos B (23)	2019	R	1998-2016	University Hospitals Leuven/Institute Jules Bordet Brussels	25	NR	NR	NR	EBRT (100%)
Mohler JL (24)	2018	P	1997-2006	CALGB (Alliance for Clinical Trials in Oncology, Statistical Center)	41	5.4	75	7	EBRT (58%)/BT (27%)/EBRT+BT (15%)
Yuh B (28)	2014	P	2004-2012	City of Hope National Cancer Center	51	NR	NR	NR	BT (43.1%)/BT+EBRT (2.0%)/EBRT (35.3%)/Proton beam therapy (11.8%)
Chade DC (30)	2011	R	1985-2009	Memorial Sloan-Kettering Cancer Center [MSKCC]/Mayo Clinic/Netherlands Cancer Institute/San Raffaele Hospital/Katholieke Universiteit [KU] Leuven/University of Sao Paulo/Vancouver General Hospital	404	NR	NR	NR	BT+EBRT (3%)/BT+EBRT+IMRT (0.5%)/BT (19%)/EBRT+ three-dimensional conformal RT (1.2%)/EBRT+IMRT (1.2%)/EBRT (63%)
Pisters LL (31)	2009	R	1990-1999	The Mayo Clinic	42	NR	NR	NR	EBRT (92.9%)/BT (7.1%)
Paparel P (32)	2009	R	1984.6-2006.9	Memorial Sloan-Kettering Cancer Center	146	NR	NR	NR	RT
Sanderson KM (35)	2006	P	1983-2002	The University of Southern California/Norris Cancer Center	51	NR	NR	NR	EBRT (57%)/Interstitial (23%)/Interstitial RT+EBRT (16%)/Proton beam (2%)
Ward JF (37)	2005	R	1967-2000	Naval Medical Center, Virginia	138	NR	NR	NR	EBRT (92%)/Interstial seeds (7%)/EBRT+seeds Less (1%)
Tefilli MV (39)	1998	R	1989.12-1995.3	Wayne State University, School of Medicine, and Barbara Ann Karmanos Cancer Center Institute	27	8.5	NR	NR	RT

R, retrospective; P, prospective; n, number; PSA, prostate specific antigen; NR, not reported; GS, Gleason score; EBRT, external beam radiotherapy; RT, radiotherapy; BT, brachytherapy; ADT, androgen deprivation therapy.

**Table 2** summarizes the disease and treatment characteristics during the peri-salvage surgery period. Across various studies, the median age at recurrence ranged from 64 to 70 years, while the median time from primary treatment to salvage therapy (TRS) varied from 36 to 82.2 months. Median pre-treatment PSA levels ranged from 3.2 to 9.1 ng/ml. Imaging modalities for pelvic recurrence included magnetic resonance imaging (MRI), computed tomography (CT), bone scan, and positron emission tomography-computed tomography (PET-CT). Pathological biopsies of recurrent lesions were performed for the majority of enrolled patients, and recurrence definitions were based on the Phoenix criteria or were not specified. Median rates for post-salvage surgery characteristics were as follows: positive surgical margins (PSM) ranged from 16% to 44%, seminal vesicle invasion (SVI) from 29.6% to 38%, lymph node involvement (LNI) from 6% to 28%, and values of pathological GS (≤7/≥8) ranged from 0.30 to 3.35. Additionally, proportions of neoadjuvant/adjuvant ADT usage and follow-up times after salvage therapy were summarized.

**Table 2:** Disease and treatment characteristics during the peri-salvage surgery period.

First author	Surgical method	Age (years, range)	Median TRS (mo, range)	Pre-salvage PSA (ng/mL, range)	Pre-salvage GS (%)			Imaging for relapse	Biopsy	BCR definition
					≤7	≥8	≤7/≥8			
Lama DJ (13)	Robot-Assisted	67.0 (63.0-71.0)	67.6(43.6-96.3)	3.7(2.4-5.8)	NR	NR	NR	Bone scan/CT/PSMA/PET	YES	Phoenix
Ribeiro L (17)	Robot-Assisted (82%)/Laparoscopic (9%)/Open (9%)	66 (62-70)	NR	7.0 (0.06-34.0)	56	44	1.27	MRI/bone scan/CT	YES	NR
Rajwa P (44)	Open	69 (64-72)	NR	3.8 (2.1-6.5)	71	29	2.45	NR	YES	Phoenix
Nathan A (18)	Robot-Assisted	70 (63-73)	NR	5.5 (3.65-11.00)	61.3	38.8	1.58	MRI/bone scan/CT/prostatespecific membrane antigen positron emission tomography scans	YES	NR
Martinez PF (open) (19)	Open	64.5(60-68)	NR	6.1 (4-7.9)	54	46	1.17	NR	YES	NR
Martinez PF (robot) (19)	Robot-Assisted		NR	6.5 (4.2-10.3)	34.6	65.4	0.53	NR	YES	NR
Marra G (20)	Robot-Assisted (47.8%)/Open (52.2%)	66 (62−70)	62.4(34.8−90)	4.2 (2.5−7.3)	55.5	35.3	1.57	NR	95.6%YES	Phoenix
Quhal F (43)	Open	69 (64-72)	NR	3.8 (2.05-6.5)	71	29	2.45	NR	YES	Phoenix
Onol FF (22)	Robot-Assisted	65.36	82.2	4.5 ± 3.28	52.1	38.3	1.36	MRI	YES	NR
Devos B (23)	Robot-Assisted (8%)/Open (92%)	69.2 (54.0-80.0)	NR	3.2 (0.2-11.4)	48	37	1.3	CT/bone scan/PET-CT/MRI	YES	Phoenix
Mohler JL (24)	Open	64 (59-68)	64	4.1(2.4-8.4)	77	24	3.21	NR	YES	NR
Yuh B (28)	Robot-Assisted	68 (63-71)	68 (40-96)	5.3 (3.2-7.44)	NR	NR	NR	CT/bone scan/Prostascint	YES	Phoenix
Chade DC (30)	Open	65	41(27-58)	4.5 (2.5-7.4)	54	20	2.7	NR	YES	NR
Pisters LL (31)	Open	67.5 (54.0-76.0)	NR	4.0 (0.3-9.6)	83.3	9.5	8.77	NR	YES	NR
Paparel P (32)	Open	65 (61-69)	55.2(32.4-72.0)	5.1 (2.7-8.9)	76	11	6.91	MRI	YES	NR
Sanderson KM (35)	Open	65(51-77)	62.4(6-224.4)	5.1-10.0	NR	NR	NR	NR	YES	NR
Ward JF (37)	Open	65.4	36	5.2	NR	NR	NR	NR	YES	NR
Tefilli MV (39)	Open	NR	39.4	9.1	62.9	37	1.7	bone scan/CT	YES	NR
First author	Patients in RFS curves (n)	Positive surgical margins (%)	Seminal vesicle invasion (%)	Lymph node involvement (%)	Pathological GS (%)			Neoadjuvant ADT (%)	Adjuvant ADT (%)	Follow-up (mo, range)
					≤7	≥8	≤7/≥8			
Lama DJ (13)	57	29	NR	6	54	23	2.35	24	22	121.1(69.6-148.8)
Ribeiro L (17)	82	37	NR	NR	62.2	41.1	1.51	NR	NR	29.5(1.6-139.3)
Rajwa P (44)	214	20	NR	NR	59.5	40.1	1.48	NR	0	25.3(15-28.5)
Nathan A (18)	49	34.7	NR	12.2	73.5	26.5	2.77	NR	36.7	16.3(8.7-35.1)
Martinez PF (open) (19)	50	30	NR	NR	36	64	0.56	43.4	NR	47(18.5-81)
Martinez PF (robot) (19)	26	26.9	NR	NR	23.1	76.9	0.30		NR	47(18.5-81)
Marra G (20)	355	29.7	NR	16	60.4	39.6	1.53	NR	NR	36 (20.4−60.5)
Quhal F (43)	214	20.1	31.4	18.7	59.8	40.2	1.49	NR	0	25.3(15-28.5)
Onol FF (22)	94	17	NR	NR	40.4	42.6	0.95	25.5	NR	32 ± 24.21
Devos B (23)	25	44	NR	28	40	48	0.83	36	0	43
Mohler JL (24)	40	17	32	12	77	23	3.35	NR	NR	91(63-104)
Yuh B (28)	51	31.4	NR	6	60.7	21.6	2.81	19.6	4	36
Chade DC (30)	404	25	30	16	51	24	2.13	NR	NR	52.8
Pisters LL (31)	42	NR	NR		NR	NR	NR	NR	NR	93.6 (20.4-184.8)
Paparel P (32)	146	16	38	13	67	20	3.35	NR	NR	45.6
Sanderson KM (35)	43	36	NR	16	56	44	1.27	18	37	86.4(108-242.4)
Ward JF (37)	137	26	NR	NR	73	27	2.70	23	56	76.8
Tefilli MV (39)	27	18.5	29.6	NR	NR	NR	NR	0	0	34.3 (13-59)

NR, not reported; TRS, time from primary treatment to salvage therapy; mo, months; PSA, prostate specific antigen; GS, Gleason score; BCR, biochemical recurrence; MRI, magnetic resonance imaging; PET-CT, positron emission tomography; CT, computed tomography; RFS, Recurrence-free survival; ADT, androgen deprivation therapy.

### Reconstructed RFS curves for the total patients

**Figure 1:**
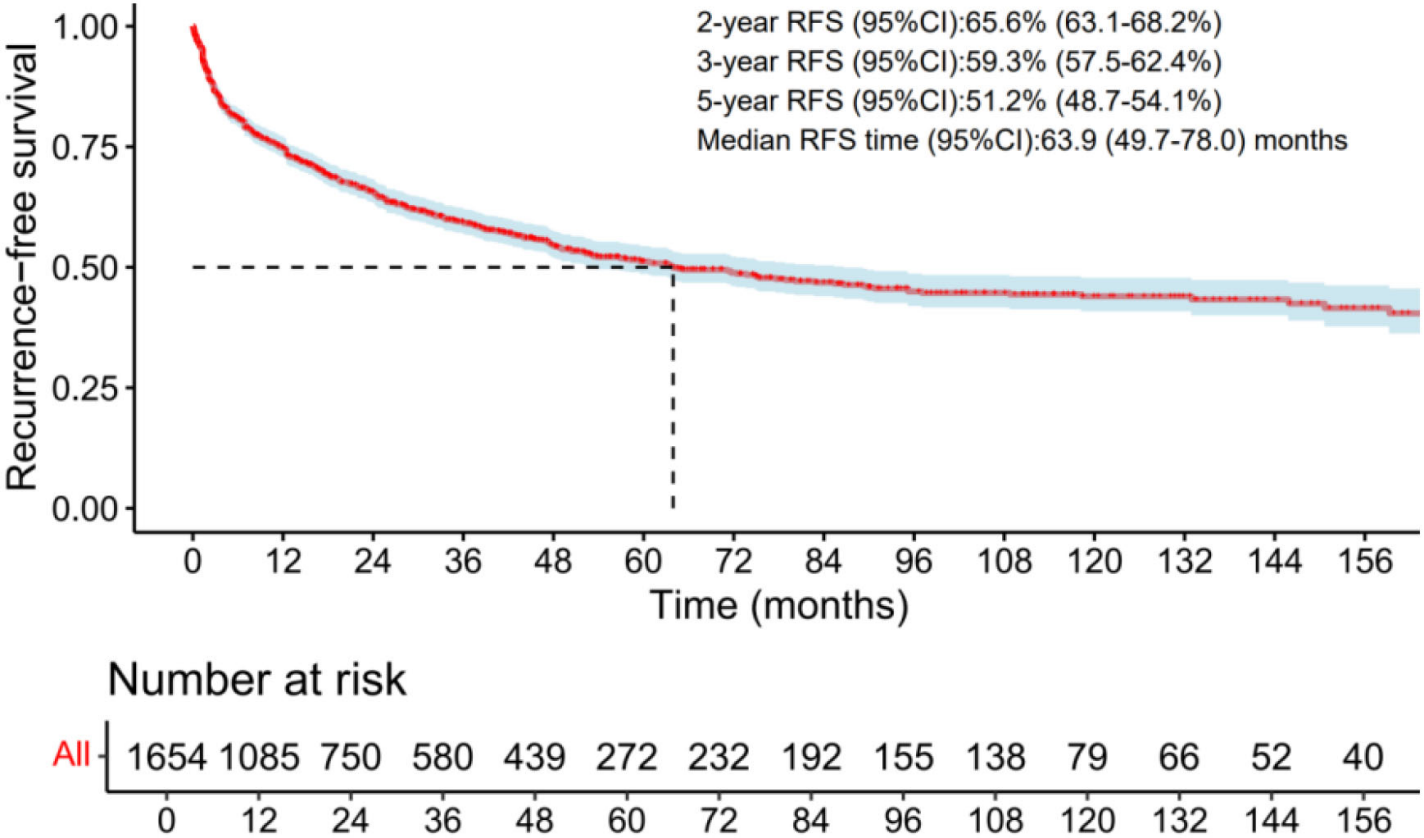
The RFS curves of the radio-recurrent patients treated with salvage surgery in the total group.

Reconstructed RFS curves for the total patients encompassed 1593 patients from 14 studies (14, 18–21, 23–25, 29, 31, 36, 38, 40, 44), with a median RFS time of 63.9 months (49.7–78.0 months). The pooled 2-, 3-, and 5-RFS rates were 65.6% (63.1-68.2%), 59.3% (57.5-62.4%), 51.2% (48.7-54.1%), respectively (**Figure 1**).

### Various subgroups for RFS

**Figure 2:**
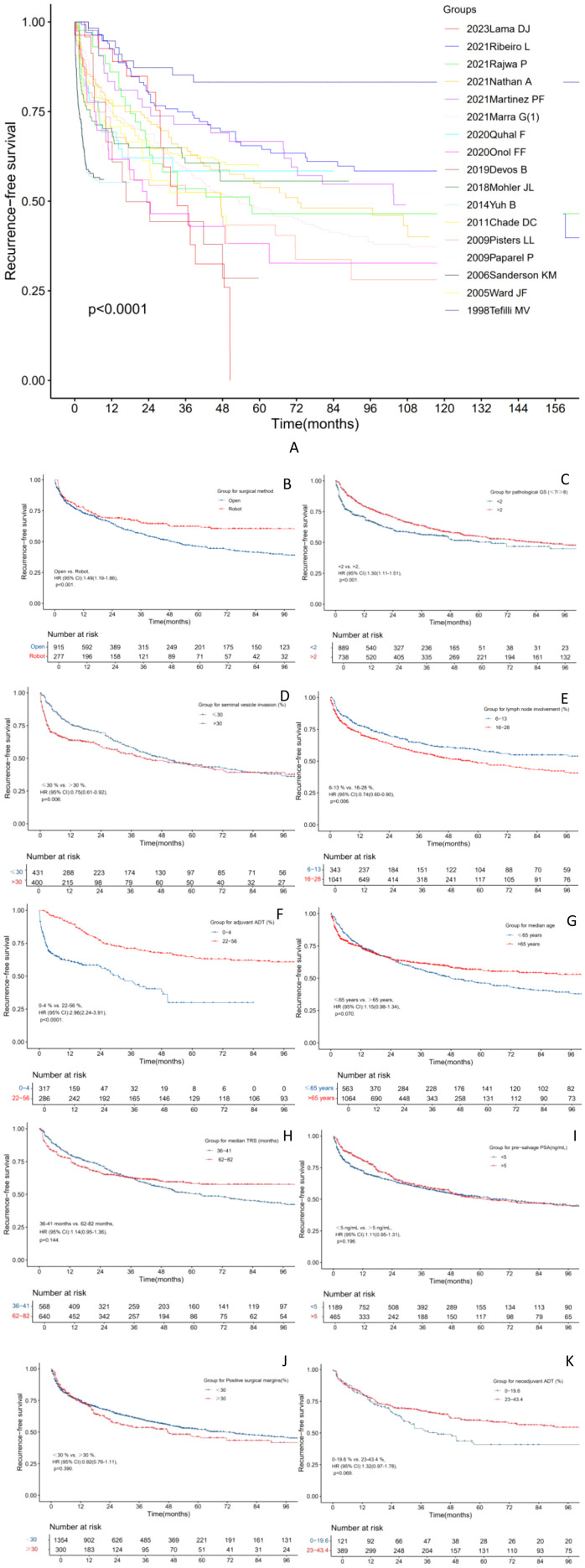
The RFS curves of the radio-recurrent patients treated with salvage surgery in different subgroups. **(A)** Grouping of different papers. **(B)** Grouping of different surgical method. **(C)** Grouping of different pathological values of Gleason Score (GS) ≤7/≥8. **(D)** Grouping of different median proportion of seminal vesicle invasion after surgery. **(E)** Grouping of different median proportion of lymph node involvement after surgery. **(F)** Grouping of different median proportion of adjuvant androgen deprivation therapy (ADT). **(G)** Grouping of different median age at time of recurrence. **(H)** Grouping of different median time from primary treatment to salvage therapy (TRS). **(I)** Grouping of different median pre-salvage prostate specific antigen (PSA) level. **(J)** Grouping of different median proportion of positive surgical margins after surgery. **(K)** Grouping of different median proportion of neoadjuvant ADT.

Subgroup analyses were conducted to evaluate various factors’ impact on RFS after salvage surgery. Initially, studies by Ribeiro L et al. (18), Martinez PF et al. (20), and Tefilli MV et al. (40) reported significantly higher RFS rates compared to studies by Devos B et al. (24), Yuh B et al. (29), and Sanderson KM et al. (36) (**Figure 2A**). Additionally, robot-assisted surgery was associated with significantly higher RFS rates compared to open surgery [open vs. robot, HR 1.49 (95% CI 1.19-1.86), p < 0.001] (**Figure 2B**). Similarly, patients with higher values of pathological GS (≤7/≥8) showed significantly higher RFS rates compared to lower values [<2 vs. >2, HR (95% CI): 1.30 (1.11-1.51), p < 0.001] (**Figure 2C**). Moreover, patients with lower rates of SVI [≤ 30% vs. >30%, HR 0.75 (95% CI 0.61-0.92), p = 0.006] and LNI [6-13% vs. 16-28%, HR 0.74 (95% CI 0.60-0.90), p = 0.006] showed notably higher RFS rates (**Figures 2D, E**). Furthermore, higher proportions of adjuvant ADT were associated with significantly higher RFS rates compared to lower proportions [0-4% vs. 22-56%, HR 2.96 (95% CI 2.24-3.91), p < 0.001] (**Figure 2F**). However, no significant differences in RFS rates were observed across different age groups at recurrence (≤65 years vs. >65 years), TRS (36–41 months vs. 62–82 months), pre-salvage PSA levels (<5 ng/ml vs. >5 ng/ml), PSM rates (<30% vs. ≥30%), and proportion of neoadjuvant ADT (0-19.6% vs. 23-43.4%), as shown in **Figures 2G-K**.

### RFS rates at 2-year or 5-year from 30 papers

As shown in **Supplementary Table S2**, the range of 2-year RFS rates reported in 30 papers was from 33.5% to 91.2%, with a median of 64.3%. For the 5-year RFS rates reported in 26 papers, the range was from 23.8% to 83.2%, with a median of 54.0% (14–43).

### Pooled analysis of severe complications according to Clavien-Dindo Scale

**Figure 3:**
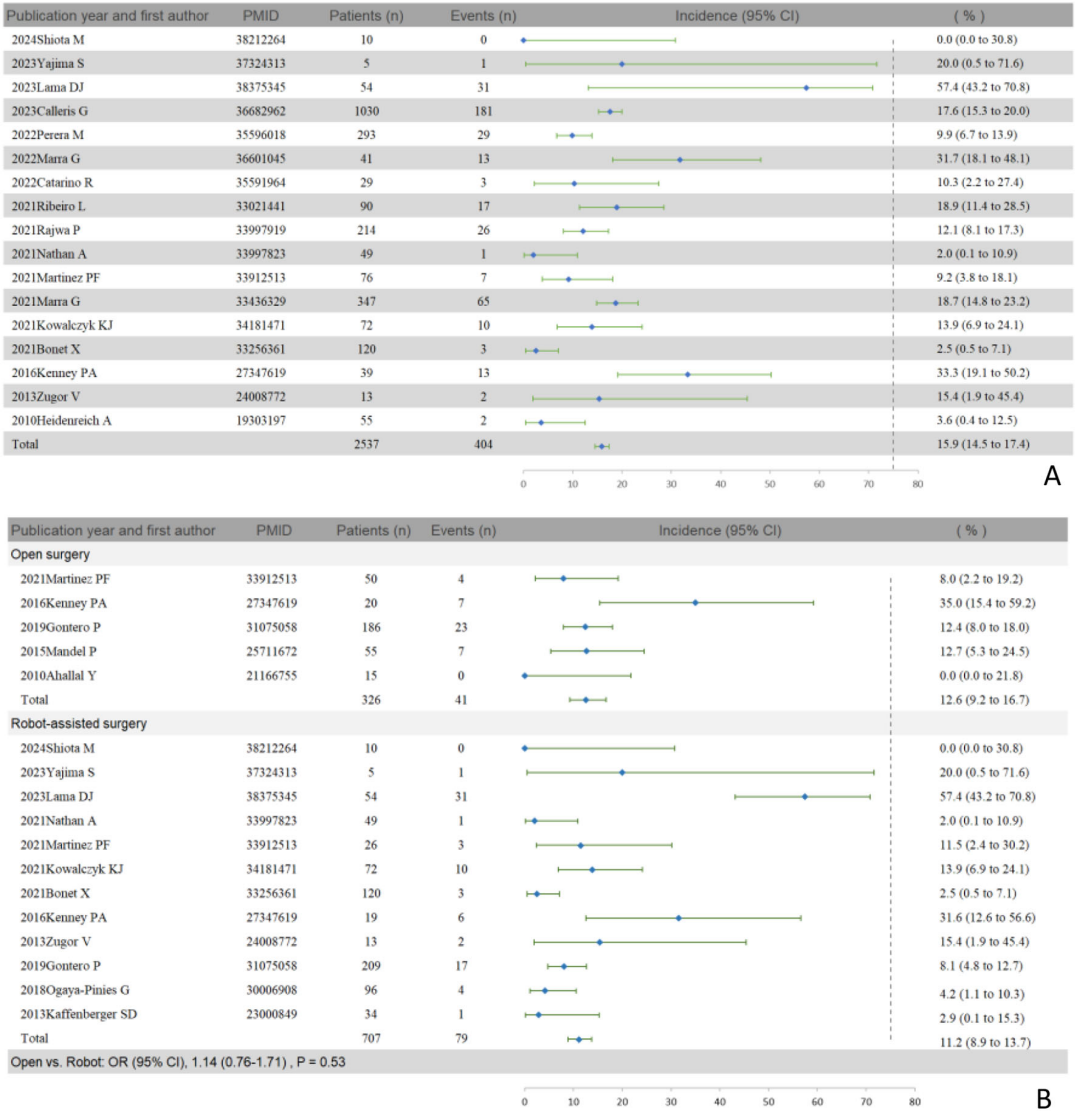
Incidences of severe complications with 95% confidence intervals (CI) across different studies based on the Clavien-Dindo Scale (CDS) after excluding duplicate data. **(A)** Total group. **(B)** Grouping of different surgical method (open vs. robot).

According to **Supplementary Table S4**, 29 papers documented severe complications. After excluding duplicated cases, a total of 22 studies (14–16, 18–22, 28, 45–57) were included in the forest plot depicting severe complications. The results indicate that out of 2532 patients, 404 (15.9%, 95% CI: 14.5 to 17.4) experienced severe complications (see **Figure 3A**). In terms of surgical methods, there was no significant difference in severe complication rates between the two methods [open vs. robot (12.6% vs. 11.2%), odds ratio (OR) (95% CI), 1.14 (0.76-1.71), p = 0.53] (see **Figure 3B**). In addition, 3 studies reported the incidence of incontinence ≥3 pads/d (the use of ≥ 3 pads per day was defined as severe incontinence) after salvage RP at 1 year (**Tables 5**). The overall incidence of severe incontinence was 17.28% (183 of 1059). 10 studies reported the incidence of ED after salvage RP at 1 year (**Supplementary Table S6**). The overall incidence was 72.76% (935 of 1285).

## Discussion

To our knowledge, this meta-analysis is the first to evaluate RFS rates (using survival curve reconstruction methods) and severe complications (using the CDS) following salvage surgery in patients with radio-recurrent prostate cancer.

Our study findings indicate that the estimated 2-year and 5-year RFS rates are slightly lower compared to Valle et al.’s meta-analysis (58). Specifically, we showed a 2-year RFS rate of 65.6% (95% CI: 63.1-68.2%), whereas Valle et al. reported 69% (95% CI: 64-74%). The 5-year RFS rate was 51.2% (95% CI: 48.7-54.1%), compared to Valle et al.’s 54% (95% CI: 49-59%). Additionally, the median 2-year RFS rate from 30 studies was 64.3% (see **Supplementary Table S2**), which is also lower than Valle et al.’s reported 69%. However, both meta-analyses reported similar data for 5-year RFS rates (both 54%). This discrepancy may stem from differences in the studies included. Our study included only 14 eligible studies for survival curves reconstruction (14, 18–21, 23–25, 29, 31, 36, 38, 40, 44), involving 1654 patients. This comprised 7 studies published after 2019 (14, 18–21, 23, 44), and excluded three studies with significant duplicate data (32, 33, 45). In contrast, Valle et al.’s study encompassed 26 studies with a total of 1439 patients, without excluding studies with substantial duplicate data and excluding those published after 2019. In summary, the principal novel contributions of our meta-analysis are threefold: (1) providing updated outcome data incorporating recent studies on robot-assisted techniques; (2) employing stricter criteria to control for patient duplication, enhancing data integrity; and (3) performing a comprehensive series of pre-specified subgroup analyses. These analyses rigorously evaluated the impact of key prognostic variables—including surgical approach, pathologic Gleason score (≤7 vs. ≥8), rates of SVI and LNI, utilization of ADT, patient age, interval to salvage therapy, pre-salvage PSA levels, and positive surgical margin rates—thereby offering new insights into factors influencing recurrence and aiding refined patient selection.

Furthermore, our study revealed lower rates of severe complications among 2537 participants across 17 studies (**Figure 3A**) compared to Valle et al.’s meta-analysis (58), which included 1617 cases from 43 studies: 15.9% (95% CI: 14.5-17.4%) vs. 21% (95% CI: 16-27%), respectively. Several factors contribute to this difference. Firstly, all of our included studies used CDS to define AEs, while Valle et al.’s studies mostly relied on descriptive records. Secondly, our inclusion criteria using the CDS did not classify severe urinary incontinence as a serious complication, whereas some studies included in Valle et al.’s analysis categorized severe urinary incontinence as a severe urological AE; given reports of severe urinary incontinence rates (≥3 pads per day) ranging from 9.7% to 35.7% (15, 16, 54), the actual incidence of severe urological AEs may exceed the aggregated values we currently report. Moreover, 14 of our 17 studies were published after 2019 (**Figure 3A**), whereas all of Valle et al.’s studies were published before 2019 (58). Lastly, we have made efforts to exclude studies with substantial duplicate data, which Valle et al. did not address. These two important factors together may impact the reported incidence rates of AEs.

In the subgroup analysis for RFS, a higher use of adjuvant ADT was linked to improved RFS outcomes [0-4% vs. 22-56%, HR 2.96 (95% CI 2.24-3.91), p < 0.001], consistent with findings from our previous meta-analysis on salvage HDR-BT (10). Furthermore, this study reinforced that lower rates of SVI and LNI, along with higher proportions of pathological GS ≤7, correlated closely with higher RFS rates. These insights may guide urologists and radiation oncologists towards more confident decisions regarding aggressive treatments like extended adjuvant ADT.

Unlike our previous meta-analysis (10), this study did not identify median age at recurrence or median TRS as prognostic factors for RFS. Notably, our meta-analysis for the first time confirmed that salvage robot-assisted surgery offers a superior RFS rate compared to salvage open surgery [open vs. robot, HR 1.49 (95% CI 1.19-1.86), p < 0.001], with similar rates of severe complications between the approaches [open vs. robot (12.6% vs. 11.2%), OR 1.14 (95% CI 0.76-1.71), p = 0.53]. Prior studies have extensively compared these surgical methods in terms of BCR rates (17, 20, 21, 51) and severe complications (20, 51, 54). However, none have shown significant differences in either BCR rates or severe complications between salvage open and robot-assisted surgeries, except for Gontero P et al.’s study (54), which suggested robot-assisted surgery might reduce anastomotic stricture (p < 0.01), blood loss (p < 0.0001), hospital stay (p < 0.0001), and improve continence outcomes (p = 0.022). However, this conclusion is subject to certain limitations. Variations in surgical timing among the included studies may be a contributing factor, as more recent cases likely benefited from newer surgical techniques and reduced operative trauma. Additionally, differences in patient age, overall tolerance, and perioperative care could also account for the observed heterogeneity in outcomes.

It is noteworthy that salvage surgery may be associated with higher rates of severe complications (15.9% for open surgery and 11.2% for robot-assisted surgery) compared to re-irradiation, particularly SBRT. Multiple retrospective studies have demonstrated the favorable safety and efficacy of salvage SBRT (59–61). In addition, not only salvage EBRT but also salvage brachytherapy is considered a safe and reliable treatment option (62). Our previous meta-analysis reported that salvage SBRT achieved a 2−year RFS rate of 64.8% with a severe genitourinary (GU) complication rate of 5.8% (63). In comparison, HDR-BT also demonstrates a similarly low rate of severe GU complications (5.8%) alongside a higher 2−year RFS of 75.9% (10). Another meta-analysis suggests that LDR-BT is associated with a similarly modest rate of severe GU complications (12.7%) and an even higher 2−year RFS of 84.6% (64).Furthermore, this study failed to confirm a significant impact of pre-salvage PSA levels on biochemical recurrence, consistent with several related studies (21, 23, 27, 28, 30, 33, 44), but differing from other findings (16, 29, 31, 35, 36, 38, 39, 45), which suggested a higher PSA level was associated with a poorer RFS rate. Similarly, this study did not substantiate a significant prognostic effect of PSM on BCR, aligning with multiple related studies (14, 18, 23, 29–31, 35, 38, 44), yet diverging from others (21, 22, 28, 36, 39), which suggested positive margins were associated with a poorer RFS rate.

While our study yields novel insights, several important limitations must be acknowledged. First and foremost, the reliability of our core findings—particularly the reconstructed survival curves and subgroup comparisons—is inherently constrained by the retrospective design and significant heterogeneity of the included studies. Variations in inclusion criteria, baseline patient characteristics (e.g., primary treatment, pre-salvage PSA, Gleason score), and perioperative management challenge the comparability of data and affect the precision of pooled estimates. In addition, while we consolidated various definitions of recurrence-free survival — such as biochemical, progression-free, and disease-free survival — for analysis, a practice common in many meta-analyses, this approach may introduce certain limitations to the conclusions. Additionally, there were significant variations in inclusion criteria, perioperative management, and baseline patient characteristics (e.g., primary treatment, surgical methods, pre-salvage age, TRS, pre-salvage PSA level, GS, PSM, SVI, LNI, pathological GS, peri-salvage ADT). These variations inevitably affect conclusions on differences in RFS among subgroups. Despite efforts to avoid duplicate data, some studies may overlap, particularly from tertiary referral centers in the US, Australia, and Europe. Furthermore, as previously mentioned, our study assessed AEs using the CDS, which does not classify severe urinary incontinence or erectile dysfunction as serious complications. Given the relatively high occurrence of severe urinary incontinence following salvage surgery, the rate of severe complications reported in our study may be lower than the actual incidence observed clinically. Lastly, discrepancies in data extraction software, survival reconstruction methods, curve resolutions, and researcher techniques may influence data restoration accuracy. Therefore, our findings’ reliability may be compromised, necessitating confirmation through relevant randomized controlled trials (RCTs).

## Conclusion

This study provides a comprehensive assessment of severe complications and conducts a pooled analysis of RFS following salvage surgery for radio-recurrent PCa. Robot-assisted surgery, lower rates of SVI and LNI, adjuvant ADT, and higher proportions of pathological GS ≤7 show promise as prognostic factors for RFS. However, confirming these findings will require RCTs due to the low levels of evidence, heterogeneity across studies, and varied RFS definitions.
