# Supplementary material for: Evaluation of the safety and efficacy of surgery for radio-recurrent prostate cancer: a systematic review and meta-analysis
Source: Front Oncol. 2026 Jan 16;15:1674005. doi: 10.3389/fonc.2025.1674005 (PMC12855099; doi:10.3389/fonc.2025.1674005)
Supplement: Supplementary file 1 [file Table1.docx]

**Supplementary Figure 1. Flow chart of literature search.**

Potential records in PubMed (n=4304)

Records excluded by inclusion and exclusion criteria (n=8934)

Abstracts (n=2319)

Records excluded by abstract review (n=2186)

No severe complications using Clavien-Dindo Scale or RFS curve reported (n=42)

Not radio-recurrent prostate cancer (n=29)

Duplicated or insufficient data about total severe complications (n=15)

RFS curves didn't show rates beyond two years (n=3)

Forty-four studies were included, with 17 studies participating in survival reconstruction and 17 studies participating in evaluation of severe complications.

Potential records in EMBASE (n=9838)

Records after duplication and removal (n=11253)

**Identification**

**Screening**

**Eligibility**

**included**

Highly relevant articles assessed for eligibility (n=133)

**Supplementary Table 1. Search strategy and results in English from their inception to July 23, 2024.**

| Database | Search query | Results |
| --- | --- | --- |
| Pubmed | ((((((((((((radiation therapy) OR (radiotherapy)) OR (radiation therapies)) OR (robot)) OR (radio-resistant)) OR (brachytherapies)) OR (radioresistant)) OR (radio-recurrent)) OR (brachytherapy)) OR (radiorecurrent)) AND (((prostatectomy[Title/Abstract]) OR (SALVAGE SURGERY[Title/Abstract])) OR (prostatectomies[Title/Abstract]))) AND (((((((((Salvage[Title/Abstract]) OR (Recurrence[Title/Abstract])) OR (local Failure[Title/Abstract])) OR (Recurrent[Title/Abstract])) OR (resistant[Title/Abstract])) OR (Radiation Failure[Title/Abstract])) OR (Relapse[Title/Abstract])) OR (Recrudescence[Title/Abstract])) OR (biochemical failure[Title/Abstract]))) AND (((Prostate[Title/Abstract]) OR (prostatic[Title/Abstract]) OR (prostatectomy[Title/Abstract]))) | 4304 |
| Embase | 1. ‘radiation therapy’ OR ‘radiotherapy’ OR ‘radio-resistant’ OR ‘brachytherapies’ OR ‘radioresistant’ OR ‘radio-recurrent’ OR ‘brachytherapy’ OR ‘radiorecurrent’  2. ‘prostatectomy’ OR ‘salvage surgery’ OR ‘prostatectomies’ OR ‘robot’  3. ‘Salvage’ OR ‘Recurrence’ OR ‘local Failure’ OR ‘Recurrent’ OR ‘resistant’ OR ‘Radiation Failure’ OR ‘Relapse’ OR ‘Recrudescence’ OR ‘biochemical failure’  4. ‘Prostate’ OR ‘prostatic’  5. 1 and 2 and 3 and 4 | 9838 |

**Supplementary Table 2. The 2-year and 5-year RFS rates of different papers using curve data reading software.**

| Publication year and first author | PMID | Patients in RFS curves （n） | 2-year RFS (%) | 5-year RFS (%) |
| --- | --- | --- | --- | --- |
| 2023Lama DJ [13] | 38375345 | 57 | 87.2 | 83.2 |
| 2023Calleris G [14] | 36682962 | 1030 | Meeting the EAU criteria :68.6;  Not meeting the EUA criteria :52.5 | Meeting the EAU criteria :55.3;  Not meeting the EUA criteria: 37.1 |
| 2022Catarino R [15] | 35591964 | 29 | 64.7 | 58.1 |
| 2021Schuetz V [16] | 35011942 | 53 | Robotic surgery:78.4; open surgery:38.1 | Robotic surgery: NR; open surgery:23.8 |
| 2021Ribeiro L [17] | 33021441 | 82 | 61.7 | NR |
| 2021Nathan A [18] | 33997823 | 49 | 63.9 | 57.7 |
| 2021Martinez PF [19] | 33912513 | 76 | Open approach:54.2; Robotic approach:61.7 | Open approach:43.7; Robotic approach:43.7 |
| 2021Marra G [20] | 33436329 | 355 | 65.2 | 58.8 |
| 2021Bonet X [21] | 33256361 | 120 | 64.3 | 55.3 |
| 2020Onol FF [22] | 31430422 | 94 | 64.7 | 55.6 |
| 2019Devos B [23] | 30666400 | 25 | 49.8 | 25.8 |
| 2018Mohler JL [24] | 30385835 | 40 | 49.3 | 38.1 |
| 2018Bonet X [25] | 30126045 | 80 | G-NS:66.1; P-NS:77.3 | NR |
| 2017Metcalfe MJ [26] | 28761588 | 70 | CTE:33.5; PTE:49.7; NTE:61.7 | CTE:26.7; PTE:0; NTE:45.3 |
| 2015Mandel P [27] | 25711672 | 55 | 54.3 | 48.8 |
| 2014Yuh B [28] | 24314031 | 51 | 61.6 | 57.7 |
| 2011Gorin MA [29] | 24578882 | 24 | 53.9 | 39.2 |
| 2011Chade DC [30] | 21420229 | 404 | 67.4 | 44.6 |
| 2009Pisters LL [31] | 19524984 | 100 | 71.2 | 60.9 |
| 2009Paparel P [32] | 18639970 | 146 | 71.5 | 54.0 |
| 2009Boris RS [33] | 19076132 | 11 | 80.4 | NR |
| 2007van der Poel HG [34] | 16949728 | 27 | 48.9 | 36.8 |
| 2006Sanderson KM [35] | 17070244 | 43 | 60.8 | 46.8 |
| 2006Darras J [36] | 16815663 | 11 | 91.2 | 61.3 |
| 2005Ward JF [37] | 15758726 | 137 | 80.8 | 65.6 |
| 2005Bianco FJ Jr [38] | 15890586 | 100 | 66.1 | 54.9 |
| 1998Tefilli MV [39] | 9697786 | 25 | 80.5 | NR |
| 1998Garzotto M [40] | 9474190 | 29 | NHT:80.1; AD tailure:49.7 | NHT:75.4; AD tailure:33.4 |
| 1995Rogers E [41] | 7526002 | 39 | 73.2 | 55.4 |
| 1995Lerner SE [42] | 7543608 | 132 | RRP:80.2; AE:42.6; TE:NR | RRP:64.7; AE:23.9; TE:NR |
| Median (range) | - | - | 64.3 (33.5-91.2) | 54.0 (23.8-83.2) |

RFS, Recurrence-free survival; NR, not reported; EAU, European Association of Urology; NS, nerve sparing; G-NS, Good NS; P-NS, Poor-NS; CTE, carcinoma with treatment effect; NTE, no treatment effect; PTE, partial treatment effect; NHT, neoadjuvant hormonal therapy; AD, androgen deprivation; RRP, radical retropubic prostatectomy; AE, anterior exenteration; TE, total exenteration.

**Supplementary** **Table 3. Details of RFS curves reconstruction for subgroups.**

| First author | Publication year | Patients in RFS curves （n） | Inclusion time | Institutions | RFS curves reconstruction or not with the influence of duplicate cases eliminated in different groups | | | | | | | | | | | | |
| --- | --- | --- | --- | --- | --- | --- | --- | --- | --- | --- | --- | --- | --- | --- | --- | --- | --- |
|  |  |  |  |  | Single | All | Surgical method | Age at recurrence | Median TRS | Pre-salvage PSA | Pre-salvage GS≤7/≥8 | Positive surgical margins | Seminal vesicle invasion | Lymph node involvement | Pathological GS ≤7/≥8 | Neoadjuvant ADT | Adjuvant ADT |
| Lama DJ [13] | 2023 | 57 | 2004-2021 | Prospective database (approval #00149) | Yes | Yes | Yes | Yes | Yes | Yes | No | Yes | No | Yes | Yes | Yes | Yes |
| Ribeiro L [17] | 2021 | 82 | 2007-2019 | Guy’s Hospital (London, UK), Institut Mutualiste Montsouris (Paris, France), Imperial College Healthcare Trust (London, UK) and The Peter MacCallum Cancer Centre (Melbourne, Australia) | Yes | Yes | No | Yes | No | Yes | Yes | Yes | No | No | Yes | No | No |
| Rajwa P* [44] | 2021 | 214 | 2007-2015 | Medical University of Vienna/King Fahad Specialist Hospital/Jordan University Hospital/ University Medical Center Hamburg-Eppendorf/Jikei University School of Medicine | Yes | No | No | No | No | No | No | No | No | No | No | No | No |
| Nathan A [18] | 2021 | 49 | 2012.1-2020.3 | University College London Hospitals NHS Foundation Trust | Yes | Yes | Yes | Yes | No | Yes | Yes | Yes | No | Yes | Yes | No | Yes |
| Martinez PF (open) [19] | 2021 | 50 | 2004.8-2019.3 | Hospital Italiano de Buenos Aires, Argentina | Yes | Yes | Yes | Yes | No | Yes | Yes | Yes | No | No | Yes | Yes | No |
| Martinez PF (robot) [19] | 2021 | 26 | 2004.8-2019.3 | Hospital Italiano de Buenos Aires, Argentina | Yes | Yes | Yes | Yes | No | Yes | Yes | Yes | No | No | Yes | Yes | No |
| Marra G [20] | 2021 | 355 | 2000-2016 | 18 tertiary referral centers in United States, Australia and Europe | Yes | Yes | No | Yes | Yes | Yes | Yes | Yes | No | Yes | Yes | No | No |
| Quhal F [43] | 2020 | 214 | 2007-2015 | Medical University of Vienna/King Fahad Specialist Hospital/Jordan University Hospital/ University Medical Center Hamburg-Eppendorf/Jikei University School of Medicine | Yes | Yes | Yes | Yes | No | Yes | Yes | Yes | Yes | Yes | Yes | No | Yes |
| Onol FF [22] | 2020 | 94 | 2008-2018 | Advent Health Global Robotics Institute | Yes | Yes | Yes | Yes | Yes | Yes | Yes | Yes | No | No | Yes | Yes | No |
| Devos B [23] | 2019 | 25 | 1998-2016 | University Hospitals Leuven/Institute Jules Bordet Brussels | Yes | Yes | No | Yes | No | Yes | Yes | Yes | No | Yes | Yes | Yes | Yes |
| Mohler JL [24] | 2018 | 40 | 1997-2006 | CALGB (Alliance for Clinical Trials in Oncology, Statistical Center) | Yes | Yes | Yes | Yes | Yes | Yes | Yes | Yes | Yes | Yes | Yes | No | No |
| Yuh B [28] | 2014 | 51 | 2004-2012 | City of Hope National Cancer Center | Yes | Yes | Yes | Yes | Yes | Yes | No | Yes | No | Yes | Yes | Yes | Yes |
| Chade DC [30] | 2011 | 404 | 1985-2009 | Memorial Sloan-Kettering Cancer Center/ Mayo Clinic/Netherlands Cancer Institute/San Raffaele Hospital/Katholieke Universiteit Leuven/University of Sao Paulo/Vancouver General Hospital | Yes | Yes | Yes | Yes | Yes | Yes | Yes | Yes | Yes | Yes | Yes | No | No |
| Pisters LL* [31] | 2009 | 42 | 1990-1999 | the Mayo Clinic | Yes | No | No | No | No | No | No | No | No | No | No | No | No |
| Paparel P* [32] | 2009 | 146 | 1984.6-2006.9 | Memorial Sloan-Kettering Cancer Center | Yes | No | No | No | No | No | No | No | No | No | No | No | No |
| Sanderson KM [35] | 2006 | 43 | 1983-2002 | the University of Southern California/Norris Cancer Center | Yes | Yes | Yes | Yes | Yes | Yes | No | Yes | No | Yes | Yes | Yes | Yes |
| Ward JF [37] | 2005 | 137 | 1967-2000 | Naval Medical Center, Virginia | Yes | Yes | Yes | Yes | Yes | Yes | No | Yes | No | No | Yes | Yes | Yes |
| Tefilli MV [39] | 1998 | 27 | 1989.12-1995.3 | Wayne State University, School of Medicine, and Barbara Ann Karmanos Cancer Center Institute | Yes | Yes | Yes | No | Yes | Yes | Yes | Yes | Yes | No | No | Yes | Yes |

* Studies contains many duplicated data with other studies. RFS, Recurrence-free survival; TRS, time from primary treatment to salvage therapy; PSA, prostate specific antigen; GS, Gleason score; ADT, androgen deprivation therapy.

**Supplementary Table 4. Details from studies documenting total severe complications according to the Clavien-Dindo Scale (CDS).**

| Publication year and first author | PMID | Time of enrollment | Surgical method | Institutions of enrollment | Patients (n) | Events (n) | Excluded for duplicate cases |
| --- | --- | --- | --- | --- | --- | --- | --- |
| 2024Shiota M [45] | 38212264 | 2020-2023 | Robot | Japan, Kyushu University Hospital | 10 | 0 | No |
| 2023Yajima S [46] | 37324313 | 2019-2021 | Robot | Japan, National Cancer Center Hospital East | 5 | 1 | No |
| 2023Lama DJ [13] | 38375345 | 2004-2021 | Robot | USA, City of Hope Comprehensive Cancer Center | 54 | 31 | No |
| 2023Calleris G [14] | 36682962 | 2000-2021 | Open (65.5%)/Laparoscopic (0.9%)/Robot (33.5%) | 14 referral centers | 1030 | 369 | No |
| 2022Perera M [47] | 35596018 | 1985-2019 | Open (79%)/Laparoscopic (21%)/Robot (5%) | USA/Spain/Switzerland, Memorial Sloan Kettering Cancer Center/Hospital Clínic de Barcelona/Reseau Hospitalier Neuchatelois | 293 | 29 | No |
| 2022Marra G [48] | 36601045 | 2007 -2021 | Open | Molinette Hospital | 41 | 13 | No |
| 2022Catarino R [15] | 35591964 | 2007.1-2019.9 | Laparoscopic | Spain, Pedro Hispano Hospital | 29 | 3 | No |
| 2021Ribeiro L [17] | 33021441 | 2007.4-2018.9 | Robot (82%)/Laparoscopic (9%)/Open (9%) | Guy’s Hospital (London, UK), Institut Mutualiste Montsouris (Paris, France), Imperial College Healthcare Trust (London, UK) and The Peter MacCallum Cancer Centre (Melbourne, Australia) | 90 | 17 | No |
| 2021Rajwa P [44] | 33997919 | 2007-2015 | NR | Austria/Poland/Germany/Saudi Arabia/Japan, Comprehensive Cancer Center/Medical University of Silesia/University Medical Center Hamburg-Eppendorf/King Fahad Specialist Hospital/The Jikei University School of Medicine | 214 | 26 | No |
| 2021Nathan A [18] | 33997823 | 2012.1-2020.3 | Robot | UK, University College London Hospitals NHS Foundation Trust | 49 | 1 | No |
| 2021Martinez PF [19] | 33912513 | 2004.8-2019.3 | Open (65.8%) | Argentina, Hospital Italiano de Buenos Aires | 50 | 4 | No |
| 2021Martinez PF [19] | 33912513 | 2004.8-2019.3 | Robot (34.2%) | Argentina, Hospital Italiano de Buenos Aires | 26 | 3 | No |
| 2021Marra G [20] | 33436329 | 2000-2016 | Open (52.2%)/Robot (47.8%) | 18 tertiary referral centers in United States, Australia and Europe | 347 | 65 | No |
| 2021Kowalczyk KJ [49] | 34181471 | 2011-2020 | Robot | USA, MedStar Georgetown University Hospital and Washington Hospital Center/Medical College of Georgia-Augusta University/UZAd University Hospital/University Hospitals Leuven/Medical College of Wisconsin/ASST Grande Ospedale Metropolitano Niguarda/Rutgers Cancer Institute of New Jersey//Weill Cornell Medicine | 72 | 10 | No |
| 2021Bonet X [21] | 33256361 | 2008.7-2018.5 | Robot | Spain/Italy, Bellvitge University hospital/advent health global robotics institute/rey Juan Carlos University hospital/University of Modena and reggio emilia | 120 | 3 | No |
| 2016Kenney PA [50] | 27347619 | 2007-2011 | Open (48.7%) | USA, Yale School/Najaj University/West Virginia University/Massachusetts General Hospital/MD Anderson Cancer Center | 20 | 7 | No |
| 2016Kenney PA [50] | 27347619 | 2007-2011 | Robot (51.3%) | USA, Yale School/Najaj University/West Virginia University/Massachusetts General Hospital/MD Anderson Cancer Center | 19 | 6 | No |
| 2013Zugor V [51] | 24008772 | 2006.2-2011.7 | Robot | Germany/Greece, Prostate Center Northwest, St. Antonius Hospital/RWTH Aachen University Hospital/Interbalkan Medical Center | 13 | 2 | No |
| 2010Heidenreich A [52] | 19303197 | 2004.1-2008.8 | Open | Germany, University of Cologne/RWTH University Aachen | 55 | 2 | No |
| 2023Marra G | 37704503 | 2000.2-2021.1 | Open (74%)/Robot (26%) | 14 referral centers | 184 | 25 | Yes |
| 2021Marra G | 32414618 | 2001-2014 | Open (91.3%)/Robot (8.7%) | San Giovanni Battista Hospital, Italy/Fundaciò Puigvert,Spain/Leuven University Hospitals,Belgium/Clinique Saint Augustin, France/Mayo Clinic, USA;/fMartini-Klinik Prostate Cancer Center, Germany/University Hospital Hamburg-Eppendorf,Germany/Netherlands Cancer Institute,Netherlands/Amsterdam University Medical Centers,Netherlands | 20 | 10 | Yes |
| 2021Bozkurt Y | 34462255 | NR | Robot | USA, University of Texas MD Anderson Cancer Center (MDACC) | 10 | 5 | Yes |
| 2020Onol FF [22] | 31430422 | 2008-2018 | Robot | USA/Italy, Advent Health Global Robotics Institute/University of Florida/University of Modena and Reggio Emilia | 93 | 4 | Yes |
| 2019Devos B [23] | 30666400 | 1998-2016 | Open (92%)/Robot (8%) | Belgium University Hospitals Leuven/Institute Jules Bordet Brussels | 25 | 19 | Yes |
| 2019Gontero P [53] | 31075058 | 2000-2016 | Open (47.1%) | 18 tertiary referral centers | 186 | 23 | Partially yes* |
| 2019Gontero P [53] | 31075058 | 2000-2016 | Robot (52.9%) | 18 tertiary referral centers | 209 | 17 | Partially yes* |
| 2018Bonet X [25] | 30126045 | 2008.1-2016.3 | Robot | Spain/USA, Hospital Universitari de Bellvitge, Barcelona/Global Robotic Institute – Florida Hospital, Celebration, FL | 80 | 0 | Yes |
| 2018Ogaya-Pinies G [54] | 30006908 | 2001.1-2016.4 | Robot | USA/France, GRI/Montsouris | 96 | 4 | Partially yes* |
| 2015Mandel P [27] | 25711672 | 2007.1-2012.12 | Open | Germany, Martini-Clinic Prostate Cancer Center | 55 | 7 | Partially yes* |
| 2014Yuh B [29] | 24314031 | 2004-2012 | Robot | USA/Italy, City of Hope National Cancer Center/University of Padua | 51 | 22 | Yes |
| 2013Kaffenberger SD [55] | 23000849 | 2006-2011 | Robot | USA, Vanderbilt University | 34 | 1 | Partially yes* |
| 2010Ahallal Y [56] | 21166755 | 2004-2010 | Open | USA, Memorial Sloan-Kettering Cancer Center | 15 | 0 | Partially yes* |

* The study was included in the pooled analysis of severe complications specifically for either the pure robot-assisted surgery group or the pure open surgery group, without being included in the summary of the total group (regardless of whether it was robot-assisted or open method).

| Publication year and first author | Surgical method | PMID | Time of enrollment | Institutions of enrollment | Patients(n) | Incontinence (1 year),≥3 pads/d | Incidence (95% CI),≥3 pads/d |
| --- | --- | --- | --- | --- | --- | --- | --- |
| 2023Calleris G | Open (65.5%)/  Laparoscopic (0.9%)/Robot (33.5%) | 36682962 | 2000-2021 | 14 tertiary referral centers | 749 | 100 | 13.35% (11.00-16.00%) |
| 2022Catarino R | Laparoscopic | 35591964 | 2007.1-2019.9 | Pedro Hispano Hospital | 28 | 10 | 35.71% (18.64-55.93%) |
| 2019Gontero P | Robot | 31075058 | 2000-2016 | 18 tertiary institutions | 162 | 32 | 19.75% (13.92-26.73%) |
| 2019Gontero P | Open | 31075058 | 2000-2016 | 18 tertiary institutions | 120 | 41 | 34.17% (25.76-43.38%) |
| Total |  |  |  |  | 1031 | 183 | 17.28% |

**Supplementary table 5 Summary of severe incontinence after salvage RP 1 year.**

**Supplementary** **table 6 Summary of erectile dysfunction after salvage RP 1 year.**

| Publication year and first author | Surgical method | PMID | Time of enrollment | Institutions of enrollment | Patients (n) | Erectile dysfunction (1 year) | Incidence (95% CI) |
| --- | --- | --- | --- | --- | --- | --- | --- |
| 2023Yajima S | Robot | 37324313 | 2019-2021 | National Cancer Center Hospital East | 5 | 5 | 100% (47.82-100%) |
| 2023Calleris G | Open (65.5%)/Laparoscopic (0.9%)/Robot (33.5%) | 36682962 | 2000-2021 | 14 tertiary referral centers | 671 | 389 | 57.97% (54.14-61.74%) |
| 2022Perera M | Open (79%)/Laparoscopic (15%)/Robot (6%) | 35596018 | 1985-2019 | Memorial Sloan Kettering Cancer Center/Hospital Clínic de Barcelona/Reseau Hospitalier Neuchatelois | 124 | 114 | 91.94% (85.67-96.06%) |
| 2022Catarino R | Laparoscopic | 35591964 | 2007.1-2019.9 | Pedro Hispano Hospital | 7 | 6 | 85.71% (42.13-99.64%) |
| 2021Kowalczyk KJ | Robot | 34181471 | 2011-2020 | 9 centers | 72 | 64 | 88.89% (79.28-95.08%) |
| 2021Bonet X | Robot | 33256361 | 2008.7-2018.5 | Bellvitge University hospital/advent health global robotics institute/rey Juan Carlos University hospital/University of Modena and reggio emilia | 120 | 101 | 84.17% (76.38-90.19%) |
| 2020De Groote R | Robot | 32461073 | 2012.1-2018.12 | Onze Lieve Vrouw Hospital Aalst | 45 | 44 | 97.78% (88.23-99.94%) |
| 2019Gontero P | Robot | 31075058 | 2000-2016 | 18 tertiary institutions | 99 | 91 | 91.92% (84.70-96.45%) |
| 2019Gontero P | Open | 31075058 | 2000-2016 | 18 tertiary institutions | 86 | 79 | 91.86% (83.95-96.66%) |
| 2013Zugor V | Robot | 24008772 | 2006.2-2011.7 | Prostate Center Northwest/St. Antonius Hospital/RWTH Aachen University Hospital/Interbalkan Medical Center | 13 | 10 | 76.92% (46.19-94.96%) |
| 2009Seabra D | Open | 19254397 | 2005.1-2007.6 | Pio XII Foundation | 43 | 32 | 74.42% (58.83-86.48%) |
| Total |  |  |  |  | 1285 | 935 | 72.76% |
